# Comparison of metabolic changes for stone risks in 24-hour urine between non- and postmenopausal women

**DOI:** 10.1371/journal.pone.0208893

**Published:** 2019-01-24

**Authors:** Zanlin Mai, Xiaoxia Li, Chonghe Jiang, Yongda Liu, Yiwen Chen, Wenqi Wu, Guohua Zeng

**Affiliations:** 1 Department of Urology, Minimally Invasive Surgery Center, The First Affiliated Hospital of Guangzhou Medical University, Guangzhou, Guangdong, China; 2 Guangdong Key Laboratory of Urology, Guangzhou, Guangdong, China; 3 Department of Intensive Care Unit, Minimally Invasive Surgery Center, The First Affiliated Hospital of Guangzhou Medical University, Guangzhou, Guangdong, China; 4 Department of Urology, The Sixth Affiliated Hospital of Guangzhou Medical University, Qingyuan, Guangdong, China; University of Illinois College of Veterinary Medicine, UNITED STATES

## Abstract

**Background:**

To explore the differences of 24-hour urine compositions associated with urolithiasis between non- and postmenopausal females.

**Methods:**

The 24-hour urine samples of female participants were collected from May 2013 to July 2014 along with national cross-sectional study of urolithiasis among adults aged ≥18 years in China. The exclusion criteria for the participants were: serum creatinine > 133μmol/L, with urinary tract infection, gout, hyperthyroidism, malignancy, had a history of cancer, kidney stones, enterectomy, had taken thiazide diuretics, allopurinol, vitamin supplement, potassium citrate or calcium supplements during the past two weeks. The compositions associated with urinary stone in 24-hour urine were measured and compared between non-and postmenopausal women.

**Results:**

A total of 603 24-hour urine samples of female participants were analyzed. 354 women with a mean age of 52.5± 14.03 (range 19–84) years met the criteria, including 160 non-menopausal women and 194 postmenopausal women. Compared to the non-menopausal women, postmenopausal women had a lower secretion of citrate (p = 0.043), magnesium (p = 0.001) and creatinine (p = 0.001) in 24h urine. Multivariate linear regression analysis showed that the menopause status was associated with the changes in magnesium (p = 0.003) and creatinine (p = 0.002) secretion, whereas not with the changes in citrate (p = 0.402) secretion.

**Conclusions:**

Postmenopausal women have a significant lower secretion of magnesium in their 24-hour urine than non-menopausal ones. We suppose that might be associated with increased risk of urinary stone formation among postmenopausal women.

## Introduction

Menopause is defined as discontinuation of menstruation for successive 12 months, which commonly occurs at about 51.4 years old [1,2]. Considerable changes in secretion of sex steroids and pituitary hormones are well described among postmenopausal women and these variations increase the risks of several diseases such as osteoporosis, cardiovascular disorders and stroke [3–5].

Urolithiasis affects about 7% women in their lifetime and the incidence of urinary stone increases after menopause [3,6]. With the life expectancy increasing, the menopausal changes have been accounted for one third of their life. Several studies have showed a positive correlation between urolithiasis and spot urine compositions in either non- or postmenopausal women [7,8], and between spot urine variations of pre- and after estrogen treatment in postmenopausal women as well [9,10]. However, we think that 24-hour urine analysis should be a fundamental for urinary stone risk evaluation, not simply by spot urine analysis [11]. For stone metabolic evaluation, the patient should collect 24-hour urine sample while stay on a self-determined diet under normal daily conditions and should ideally be stone free for at least 20 days [12], so we only selected non-stone forming women to participate in the present study, to investigate the metabolic changes associated with urolithiasis in non- and postmenopausal women by evaluating their 24-hour urine compositions.

## Materials and methods

### Study population

From May 2013 to July 2014, we conducted a cross-sectional study to estimate the prevalence and risk factors of urolithiasis among adults aged≥18 years in China [13]. Participants underwent urinary tract ultrasonographic examinations, responded to questionnaires, and provided blood and urine samples for analysis. The standard blood analysis protocols included complete blood count, fasting serum glucose, creatinine, urea, uric acid, high-density lipoprotein, low-density lipoprotein, triglycerides, cholesterol, sodium, potassium, calcium and chloride. The estimated creatinine clearance rate (eCCr) of women was calculated by the Cockcroft-Gault equation: $\text{eCCr}\left(\text{mL}/\text{min}\right)=\frac{\left(140-\text{Age}\right)\times \text{Weight}\left(\text{kg}\right)}{\text{Cr}\left(\text{umol}/\text{L}\right)\times 0.818}\times 0.85$. A total of 9310 participants (3792 men and 5518 women) completed the cross-sectional survey. At the same time, we collected 24-hour urine samples of 603 female participants to carry out the metabolic evaluation of urolithiasis. They were non-incentivized volunteers and remained on a normal diet.

### Subject selection

Menopause is defined as discontinuation of menstruation for successive 12 months [1]. The status of non- or postmenopause was self-reported. Women with artificial menopause such as bilateral salpingo-oophorectomy and/or hysterectomy were excluded. To avoid incomplete urine collections, 24-hour urine creatinine had to be greater than 600 mg (5.3mmol). Other exclusion criteria were: with a history of cancer, kidney stones, enterectomy, gout, primary or secondary hyperparathyroidism, had high serum creatinine level (>133 umol/L), previous therapy with thiazide, allopurinol, vitamin supplements, calcitonin, potassium citrate or calcium supplements during the past two weeks. This study was approved by the Ethics Committee of the First Affiliated Hospital of Guangzhou Medical University, China (S1 File). In addition, written informed consents were obtained from all the participants.

### Collection and analysis of 24-hour urine

The method of 24-hour urine sample collections and analysis has been previously described [14]. Urine oxalate and citrate were measured by means of ion exchange chromatography (Metrohm, Switzerland). Urine sodium, potassium, chloride, calcium, phosphate, and creatinine were determined by UnicelDxC 600 synchronic biochemical detecting system. Urine urate and magnesium were measured with Beckman coulter AU680 automatic biochemistry analyzer. The pH values were measured with a glass electrode in a calibrated pH meter (Mettler Toledo, Switzerland). We also calculated standardized estimates of the ion active products of calcium oxalate, calcium phosphate (AP(CaOx) indexs and AP(CaP) indexs according to the formulas as given below [15]. In these calculations, 24-hour urine calcium, oxalate, citrate, magnesium, and phosphate were expressed in mmol and the volume in liters. In the standardized form used in these expressions the 24-hour urine volume was set to 1.5 L and the pH to 7.0.

$$AP(CaOx)index_{s}=\frac{1.9*Calcium^{0.84}*Oxalate}{Citrate^{0.22}*Magnesium^{0.12}*1.5^{1.03}}$$

$$AP(CaP)index_{s}=\frac{2.7*10^{-3}*Calcium^{1.07}*Phosphate^{0.70}*{\left(7.0-4.5\right)}^{6.8}}{Citrate^{0.20}*1.5^{1.31}}$$

All 24-hour urine samples were analyzed within 72 hours of collection. The analyses were performed in the First Affiliated Hospital of Guangzhou Medical University according to standardized protocols.

### Statistical analysis

Comparisons of the baseline characteristics between non- and postmenopausal women were performed using the Student’s t-test for continuous variables and chi-squared for categorical variables. Univariate comparisons of urinary composition between non- and postmenopausal women were accomplished using the Student’s t-test. Multivariate linear regression was adjusted for possible confounders, including body mass index (BMI), hypertension, fasting glucose, eCCr, serum uric acid, calcium, total cholesterol, high-density lipoprotein, low-density lipoprotein and triglycerides. We did not transform the non-normally distributed urinary factors because the residuals in the regression analyses approximated for normal distribution and our sample size was relatively large. P value was two-tailed and < 0.05 was considered statistically significant. The statistical analyses were performed using SPSS software (version 17.0, IBM, Armonk, USA).

## Results

**Fig 1:**
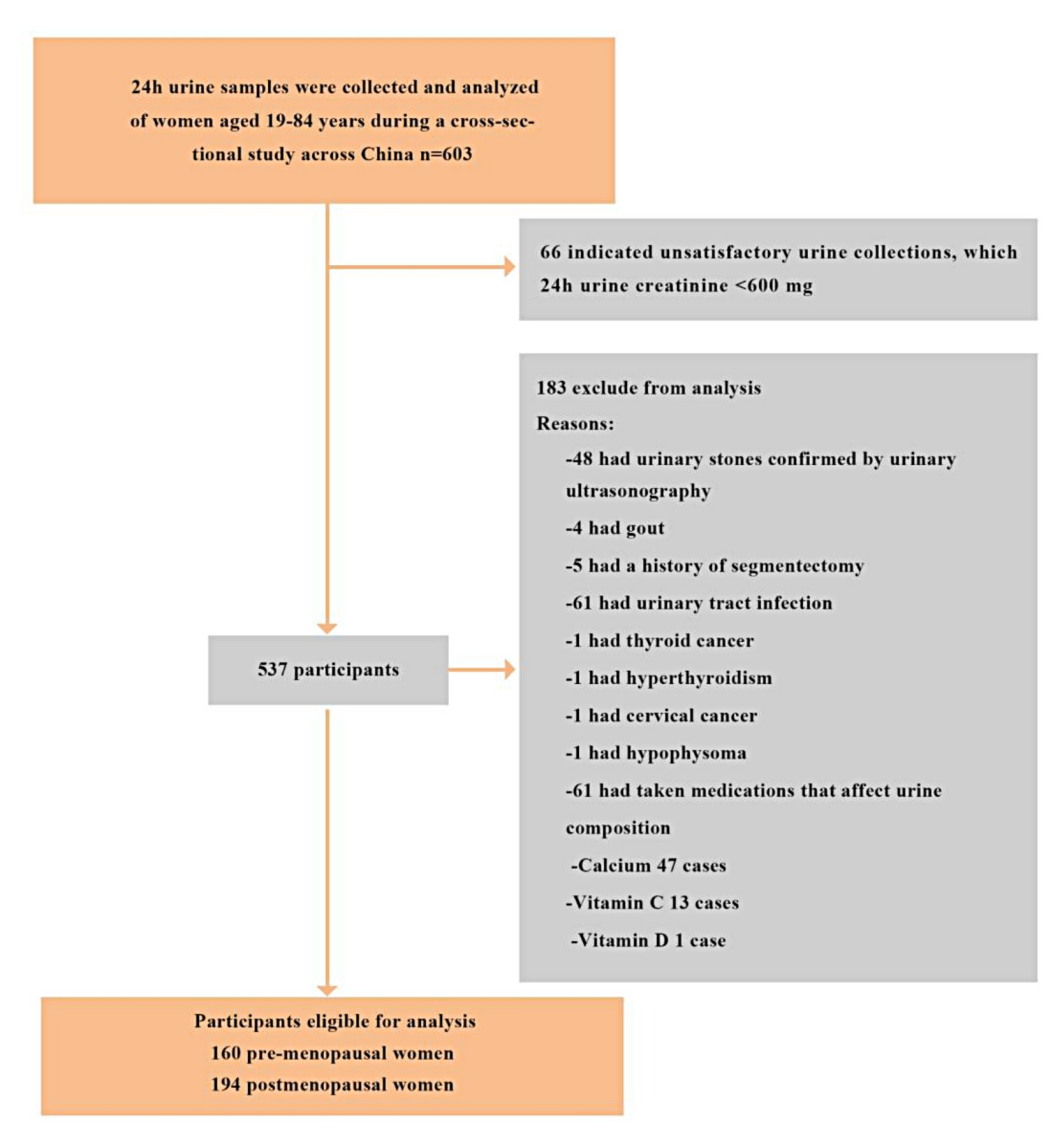
Flow chart of participants identified, included, and excluded.

A total of 603 women provided their 24-hour urine samples to be analyzed by the special trained laboratorian in our research group, 249 cases were excluded from the study based on the criteria. Leaving a total of 354 subjects with a mean age of 52.5± 14.03 (range 19–84) years met the criteria and were recruited in this study, including 160 non-menopausal women and 194 postmenopausal women (Fig 1). The distributions and characteristics of non- and postmenopausal women were shown in Table 1. As expected, the postmenopausal women showed a higher prevalence of hypertension, higher level of serum uric acid, total cholesterol, triglyceride and low-density lipoprotein, but they had lower serum creatinine and eCCr.

**Table 1:** Baseline characteristics of non- and postmenopausal women^a^.

Characteristics	Non-menopausal females (n = 160)	Postmenopausal females (n = 194)	p-value ^b^
Age, yr	39.98 ± 8.43	62.51 ± 8.44	<0.001
Weight, kg	59.42 ±10.19	55.88 ±9.16	0.001
BMI, kg/m^2^	23.76 ± 4.0	23.65 ± 3.42	0.77
Hypertension, n(%)	29(18.1)	93(47.9)	<0.001
Fasting glucose, mmol/L	5.09 ± 1.47	5.19 ± 0.89	0.45
serum creatinine, μmol/L	69.89±16.98	62.58±13.17	<0.001
eCCr, ml/min	93.66±30.43	75.16±22.60	<0.001
Serum calcium, mmol/L	2.37±0.16	2.41±0.14	0.002
Serum uric acid, μmol/L	248.77±71.36	272.84±79.49	0.003
Total cholesterol, mmol/L	4.24 ± 1.04	5.09 ± 0.97	<0.001
Triglyceride, mmol/L	1.41 ± 2.42	1.43 ± 0.70	0.942
Low-density lipoprotein, mmol/L	2.24±0.84	2.93±0.89	<0.001
High-densitylipoprotein, mmol/L	1.31±0.47	1.44±0.34	0.003

BMI, body mass index; eCCr, estimated creatinine clearance rate.

^a^ Data are presented as means ± SD.

^b^ p < 0.05 was considered statistically significant.

Analysis of 24-hour urine compositions revealed that postmenopausal females had lower excretions of citrate, creatinine, and magnesium than non-menopausal females (Table 2). The results of multivariate linear regression analysis are shown in Table 3. The differences of urinary composition between non- and postmenopausal women were compared. After adjustment for BMI, eCCr, hypertension, serum calcium, fasting glucose, uric acid, total cholesterol, high-density lipoprotein, low-density lipoprotein, triglycerides and status of menstruation, the excretions of magnesium and creatinine were significantly lower in postmenopausal females than in non-menopausal ones. No differences of other components between these two groups were observed.

**Table 2:** Univariable analysis of 24-hour urine composition between non-menopausal and postmenopausal women^a^.

Characteristics	Non-menopausal females (n = 160)	Postmenopausal females (n = 194)	p-value^b^
Calcium, mmol/24h	3.61±1.83	3.69±2.05	0.713
Oxalate, mmol/24h	0.26±0.14	0.24±0.15	0.191
Citrate, mmol/24h	2.51±1.56	2.18±1.49	0.043
Urate, mmol/24h	2.96±1.05	2.75±1.02	0.058
Sodium, mmol/24h	190.17±113.36	175.27±197.73	0.399
Potassium, mmol/24h	44.79±18.31	43.41±21.40	0.518
Magnesium, mmol/24h	3.80±1.59	3.00±1.42	<0.001
Phosphorus, mmol/24h	15.76±6.22	16.34±5.97	0.373
Chloride, mmol/24h	185.58±113.46	175.66±203.06	0.582
Creatinine, mmol/24h	8.70±2.24	7.95±1.80	<0.001
Volume, ml/24h	1373.75±630.26	1270.82±623.99	0.125
pH	6.31±0.66	6.23±0.79	0.280
AP (CaOx) Index_s_	0.69±0.51	0.67±0.53	0.779
AP (CaP) Index_s_	19.16±12.45	21.36±16.90	0.172

AP (CaOx) Index_s,_ the ion activity products of calcium oxalate; AP (CaP) Index_s,_ the ion activity products of calcium phosphate.

^a^ Data are presented as means ± SD.

^b^p < 0.05 was considered statistically significant.

**Table 3:** Multivariable adjusted analysis of 24h urine composition comparing non-menopausal women to postmenopausal women.

	Difference^a^	95%CI	p-value
Calcium, mmol/24h	0.076	(-0.245,0.774)	0.233
Oxalate, mmol/24h	-0.086	(-0.062,0.011)	0.176
Citrate, mmol/24h	-0.053	(-0.539,0.217)	0.402
Urate, mmol/24h	-0.094	(-0.454,0.063)	0.137
Sodium, mmol/24h	-0.026	(-50.781,33.690)	0.691
Potassium, mmol/24h	-0.069	(-7.798,2.262)	0.280
Magnesium, mmol/24h	-0.183	(-0.932,-0.197)	0.003
Phosphorus, mmol/24h	0.060	(-0.781,2.241)	0.343
Chloride, mmol/24h	-0.014	(-47.952,38.413)	0.828
Creatinine, mmol/24h	-0.188	(-1.263,-0.278)	0.002
Volume,ml/24h	-0.126	(-316.452,0.410)	0.049
pH	0.093	(-0.278,0.090)	0.315
AP (CaOx) Index_s_	-0.032	(-0.165,0.099)	0.623
AP (CaP) Index_s_	0.095	(-0.935,6.681)	0.139

AP (CaOx) Index_s,_ the ion activity products of calcium oxalate; AP (CaP) Index_s,_ the ion activity products of calcium phosphate.

^a^Reference to non-menopausal females.

## Discussion

The present study demonstrated that postmenopausal women had lower levels of magnesium, and creatinine in the 24-hour urine than non-menopausal women. Our subjects were volunteers from a cross-sectional study in China. All participants remained on a normal diet and fluid intake during the 24-hour urine sample collection. All the postmenopausal women were at the status of a natural biological menopause. Therefore, the outcomes would represent the changes in 24-hour urine compositions of women before and after menopause.

Magnesium is an important inhibitor of urolithiasis because it can reduce calcium oxalate crystal formation in urine [16]. It is mainly absorbed by the small intestines [17], stored inside the cells or in bone [18,19], and secreted by kidney for homeostasis. The absorptive amount of magnesium is mainly dependent on concentration of serum magnesium. The lower serum magnesium level, the more magnesium is absorbed [17]. Conversely, the re-absorption and excretion of magnesium in kidneys are influenced by several unclarified mechanisms, such as different hormone regulation [20]. It was reported that either lower or higher serum magnesium was observed among natural postmenopausal women, nevertheless, estrogen therapy could decrease its secretion in urine [21–23]. By contrast, we found that the secretion of magnesium in 24-hour urine was statistically lower among postmenopausal women than among non-menopausal women. Multivariate linear regression showed that the status of menopause was an independent factor. We proposed that there were two explanations on this opposite findings. Firstly, the spot urine sample, which was used in previous study, may not be suitable for evaluation of urinary metabolic abnormalities [11]. The drawback of spot urine is that there are variability in different time of the urine and the concentrations of contents could be different from void to void. Secondly, in addition to the estrogen, there are several other factors for magnesium regulation in kidneys, such as diet, diabetes, etc [20].

The amount of urinary creatinine secretion is one of the most specific indexes of total body muscle mass, which is related to age, BMI and meat intake [24,25]. Normally, older people have a lower urinary creatinine secretion than younger people, and increased BMI is associated with increased urinary creatinine [26]. The same trend was demonstrated by the present study, e.g. the eCCr (p<0.001), serum creatinine (p<0.001), and creatinine (p<0.001) in 24h urine, although BMI was similar between these two groups. It was reported that the urinary calcium level was higher in postmenopausal women than in non-menopausal women [8]. However, this was not the case in our study.

Reduced urinary citrate excretion is a well-known modifiable risk factor for nephrolithiasis formation [27]. Previous study showed that estrogen replacement could increase urinary citrate excretion in postmenopausal women, lead to decrease the risk of subsequent calcium stone formation [28]. In the present study, the postmenopausal women secreted significant less citrate in 24-hour urine than non-menopausal women. However, after adjustment for other variables, the contribution for these differences was not due to the status of menopause women (Table 3). Furthermore, no one had used estrogen replacement among this study cohort.

There were some limitations in the present study. Firstly, we used volunteers as the subjects of investigation instead of a randomized sample, which might have introduced a sampling bias. Secondly, we did not have the data of serum estrogen and details of nutrient intake from the subjects, which is difficult to infer the true impact of estrogen on the urinary stone formation. Since the development of urinary stone is affected by lifestyle and other health-related factors, further multicenter randomized controlled studies are required to verify the outcomes of the present study.

## Conclusions

Postmenopausal women have a significant lower secretion of magnesium in 24h urine than non-menopausal ones. We suppose that this finding might be associated with increased risk of urinary stone formation among postmenopausal women.

## Supporting information

S1 File Ethical approval form. (PDF)

S2 File Study checklist. (PDF)

S3 File Data. (XLSX)

## References

[1] Ghanchilar N, Khamene S, Shahamfar J, Jafari M. Attitude of women about menopause and it's related factors. Journal of Tabriz University of medical sciences.2005; 37:54–57.

[2] Jaszmenn LJB. Epidemiology of the climacteric syndrome, chap 2, In Campbell S(Ed): Management of the menopause and postmenopausal years. Lancaster, England, MTP Press, 1976 pp.11–23.

[3] Stamatelou KK, Francis ME, Jones CA, Nyberg LM, Curhan GC. Time trends in reported prevalence of kidney stones in the United States: 1976–1994. Kidney Int. 2003; 63:1817–1823. 10.1046/j.1523-1755.2003.00917.x 12675858

[4] Padubidri VG, Daftary SN. Shaw's Textbook of Gynecology 13th edition. New Delhi: Elsevier; Menopause, premature menopause and post menopausal bleeding; 2004 pp.56–67.

[5] Ganz PA.Breast cancer, menopause and long-term survivorship: Critical issues for the 21st century. Am J Med. 2005; 118:136–141. 10.1016/j.amjmed.2005.09.047 16414339

[6] Yasui T, Iguchi M, Suzuki S, Kohri K. Prevalence and epidemiological characteristics of urolithiasis in Japan: national trends between 1965 and 2005. Urology.2008; 71:209–213. 10.1016/j.urology.2007.09.034 18308085

[7] Curhan GC, Willett WC, Speizer FE, Stampfer MJ. Twenty-four-hour urine chemistries and the risk of kidney stones among women and men. Kidney Int. 2001; 59(6):2290–2298. 10.1046/j.1523-1755.2001.00746.x 11380833

[8] Nordin BE, Need AG, Morris HA, Horowitz M. Biochemical variables in pre- and postmenopausal women: reconciling the calcium and estrogen hypotheses. Osteoporosis Int, 1999; 9:351. 10.1007/s001980050158 10550453

[9] Dey J, Creighton A, Lindberg JS, Fuselier HA, Kok DJ, Cole FE, et al Estrogen replacement increased the citrate and calcium excretion rates in postmenopausal women with recurrent urolithiasis. J Urol. 2002; 167(1):169–171. 11743298

[10] Kramer HM, Grodstein F, Stampfer MJ, Curhan GC. Menopause and postmenopausal hormone use and risk of incident kidney stones. J Am Soc Nephrol.2003; 14(5):1272–1277. 12707395 10.1097/01.asn.0000060682.25472.c3

[11] Hong YH, Dublin N, Razack AH, Mohd MA, Husain R. Twenty-four Hour and Spot Urine Metabolic Evaluations: Correlations Versus Agreements. Urology. 2010; 75 (6):1294–1298. 10.1016/j.urology.2009.08.061 19914693

[12] Norman RW, Bath SS, Robertson WG, Peacock M. When should patients with symptomatic urinary stone disease be evaluated metabolically? J Urol.1984; 132: 1137 6502804 10.1016/s0022-5347(17)50064-6

[13] Zeng G, Mai Z, Xia S, Wang Z, Zhang K, Wang L, et al Prevalence of kidney stones in China: an ultrasonography based cross-sectional study. BJU Int. 2017; 7;120(1):109–116. 10.1111/bju.13828 28236332

[14] Zhu W, Mai Z, Qin J, Duan X, Liu Y, Zhao Z, et al Difference in 24-Hour Urine Composition between Diabetic and Non-Diabetic Adults without Nephrolithiasis. PLoS One. 2016; 2 23;11(2):e0150006 10.1371/journal.pone.0150006 26906900 PMC4764372

[15] Tiselius HG. Medical evaluation of nephrolithiasis. Endocrinol Metab Clin North Am.2002; 31:1031–50. 12474644 10.1016/s0889-8529(02)00027-0

[16] Hallson PC, Rose GA, Sulaiman S. Magnesium reduces calcium oxalate crystal formation in whole human urine. Clin Sci, 1982; 62:17–21. 7056030 10.1042/cs0620017

[17] Jahnen DJ, Ketteler M. Magnesium basics. Clin. Kidney J. 2012; 5, i3–i14. 10.1093/ndtplus/sfr163 26069819 PMC4455825

[18] Rude RK. Magnesium In Modern Nutrition in Health and Disease, 11th ed; Ross AC, Caballero B, Cousins RJ, Tucker KL, Ziegler TR, Eds. Lippincott Williams & Wilkins: Baltimore, MA, USA, 2012 pp. 159–175.

[19] Classen HG, Nowitzki S. The clinical importance of magnesium. 2. The indications for supplementation and therapy. Fortschr. Med.1990 10, 198–200. 2187780

[20] Uwe Gröber, Joachim Schmidt, Klaus Kisters. Magnesium in prevention and therapy. Nutrients.2015; 8199–8226. 10.3390/nu7095388 26404370 PMC4586582

[21] Sonu Y, Avinash SS, Sreekantha, Arun Kumar K, Malathi M, Shivashankara AR. Effect of Oestrogen on Altering the Serum and Urinary Levels of Calcium, Phosphate and Magnesium in Hysterectomised Women Compared to Natural Menopausal South Indian Women: A Case Control Study.Indian J Clin Biochem.2016; 326–331. 10.1007/s12291-015-0532-y 27382205 PMC4910850

[22] Lindsay R, Hart DM, Forrest C. Effect of a natural and artificial menopause on serum, urinary and erythrocyte magnesium.Clin Sci (Lond). 1980;255–257. 7363567 10.1042/cs0580255

[23] Aydin H, Deyneli O, Yavuz D, Gözü H, Mutlu N, Kaygusuz I, et al Short-term oral magnesium supplementation suppresses bone turnover in postmenopausal osteoporotic women. Biol Trace Elem Res.2010; 133(2):136–143. 10.1007/s12011-009-8416-8 19488681

[24] Heymsfield SB, Arteaga C, McManus C, Smith J, Moffitt S. Measurement of muscle mass in humans: validity of the 24-hour urinary creatinine method. Am J Clin Nutrl. 1983; 37: 478–494. 10.1093/ajcn/37.3.478 6829490

[25] Wyss M, Kaddurah-Daouk R. Creatine and Creatinine Metabolism. Physiol Rev. 2000; 80: 1107–1213. 10.1152/physrev.2000.80.3.1107 10893433

[26] Barr DB, Wilder LC, Caudill SP, Gonzalez AJ, Needham LL, Pirkle1 JL. Urinary Creatinine Concentrations in the U.S. Population: Implications for Urinary Biologic Monitoring Measurements. Environ Health Perspect. 2005; 113(2)192–200. 10.1289/ehp.7337 15687057 PMC1277864

[27] Nicar MJ, Skurla C, Sakhaee K, Pak CY. Low urinary citrate excretion in nephrolithiasis. Urology.1983; 21:8–14. 6823713 10.1016/0090-4295(83)90113-9

[28] Dey J, Creighton A, Lindberg JS, Fuselier HA, Kok DJ, Cole FE, et al Estrogen replacement increased the citrate and calcium excretion rates in postmenopausal women with recurrent urolithiasis. J Urol. 2002; 167:169–171. 11743298

