# Supplementary material for: Comparison of metabolic changes for stone risks in 24-hour urine between non- and postmenopausal women
Source: PLoS One. 2019 Jan 24;14(1):e0208893. doi: 10.1371/journal.pone.0208893 (PMC6345460; doi:10.1371/journal.pone.0208893)
Supplement: S1 File — (PDF) [file pone.0208893.s001.pdf]

广州医科大学附属第一医院科研项目审查伦理委员会

试验审批件

医科伦审 2012 第 31 号

项目名称	中国成年人群尿石症流行病学调查		
申办方	广州医科大学附属第一医院泌尿外科	项目负责人	曾国华

委员姓名	性别	职称/职务	单位名称	出席签名
郑劲平	男	教授	广州医科大学附属第一医院	[illegible]
刘宇平	男	研究员	广州医科大学附属第一医院	—
宋儒亮	男	律师	广东诺臣律师事务所	[illegible]
洪巧妍	女	科长	广州市越秀区人民街道办事处计生办	[illegible]
姚谦明	男	副教授	广州医科大学附属第一医院	[illegible]
黄锦坤	男	副主任医师	广州医科大学附属第一医院	[illegible]
刘桂卿	女	副教授	广州医科大学附属第一医院	[illegible]
欧阳斌	女	副主任医师	广州医科大学附属第一医院	欧阳斌
夏鑫华	男	副教授	广州医科大学附属第一医院	—
魏理	女	副主任药师	广州医科大学附属第一医院	[illegible]
谭志坚	男	高级工程师	广州医科大学附属第一医院	[illegible]

表决结果	同意<br>8 票	作必要修改后同意<br>1 票	作必要修改后重新上会<br>0 票	不同意<br>0 票	终止或暂停先前批准的试验<br>0 票	委员人数 11 人<br>出席人数 9 人<br>回避 0 人<br>弃权 0 人
申办者送审资料：	审查材料目录：<br>详细目录另附					

审查日期：2012 年 9 月 22 日	审查地点：广州医科大学附属第一医院新大楼三十楼东侧会议室
联系方式：广东省广州市沿江西路 151 号广州医科大学附属第一医院科研科	

电话：020-83062939	传真：020-83177207	联系人：余达加

科研项目审查伦理委员会审批意见：

审查项目“中国成年人群尿石症流行病学调查”。该研究由广州医科大学附属第一医院泌尿外科为临床研究负责单位。

伦理委员会经过详细审阅材料（伦理申请表、伦理审批表、临床研究方案、知情同意书等）及讨论后，按照国家有关法律法规，得出相关意见：

1. 同意批准该临床试验的实施，请严格按照已通过审查的临床试验方案进行，研究过程中确保受试者的权益。

2. 申请材料如有更新，请及时递交备案。项目结束时，请递交项目总结报告。该项目开展满一年，请向本伦理委员会提交年度研究进展报告。

主任委员签名 [illegible]

单位盖章

2012 年 9 月 22 日

声明：本伦理委员会是独立的，组成和工作程序符合 GCP 原则及国家相关法律法规。所有出席的委员均在有效任职期间。特此声明。

广州医科大学附属第一医院科研项目审查伦理委员会

试验审批件（附页）

医伦审2012第31号

项目名称	中国成年人群尿石症流行病学调查		
申办方	广州医科大学附属第一医院泌尿外科	项目负责人	曾国华

项目审查所使用的资料目录及版本号：

序号	文件名称	版本号	版本日期
1.	科研伦理审查申请表		
2.	科研伦理审批表		
3.	临床研究方案		
4.	知情同意书（中文）		

结合此前对该临床研究的伦理审批意见，现已对申办方提交的上述材料进行备案，同意批准“中国成年人群尿石症流行病学调查”的实施。

临床试验请严格按照已通过审查的临床研究方案进行，研究过程中确保受试者的权益。试验方案如有更改，请及时递交备案。

主任委员签名：[illegible]

日期：2012.5.22

Ethical approval form

Approval Form of Ethics Committee in the First Affiliated Hospital of Guangzhou Medical University					
					No.201231
Project	China National Epidemiological Study of Urolithiasis				
Sponsor	Department of urology, The First Affiliated Hospital of Guangzhou Medical University			Head of this project	Guohua Zeng
Name	Gender	Job title	Affiliation		Signature
Jinping Zhen	Men	Professor	The First Affiliated Hospital of Guangzhou Medical University		
Yuping Liu	Men	Professor	The First Affiliated Hospital of Guangzhou Medical University		
Ruliang Song	Men	Lawyer	Nuochen Law Firm in Guangdong Province		
Qiaoyuan Hong	Woman	Section chief	Family planning office, Renming Street, Yuexiu District, Guangzhou		
Qianming Yao	Men	Associate professor	The First Affiliated Hospital of Guangzhou UniversityMedical		
Guiqing Liu	Woman	Associate professor	The First Affiliated Hospital of Guangzhou Medical University		
Bing OuYang	Woman	Associate chief physician	The First Affiliated Hospital of Guangzhou Medical University		
Xinhua Xia	Men	Associate professor	The First Affiliated Hospital of Guangzhou Medical University		
Zhijian Tan	Man	Senior engineer	The First Affiliated Hospital of Guangzhou Medical University		
Voting results	Agreement 8 votes	Agreement after some necessary modification: 1 vote	Disagreement: 0 vote	Stop the trail which was approved before:0 vote	Members: 11; Be present: 9 Avoiding: 0; Give up:0
Submitted materials	List of materials: are presented in appendix				
Date of review: 22/9/2012		Review site: The meeting room in the 30th floor of The First Affiliated Hospital of Guangzhou Medical University			
Contact address	Research service office, The First Affiliated Hospital of Guangzhou Medical University, 151#, Yuanjiang Road, Guangzhou, Guangdong Province				
Tel.	020-83062939	Fax:020-83177207		Contact to: Dajia Yu	

Comments of the Ethics Committee in the First Affiliated Hospital of Guangzhou Medical University:

The Ethics Committee has reviewed the project of "China National Epidemiological Study of Urolithiasis". This project is conducted by department of urology, the First Affiliated Hospital of the Guangzhou Medical University.

After reviewing and discussing the submitted materials(including ethics application form, ethics approval form, the protocol of this study, informed consent and so on), the Ethics Committee of the First Affiliated Hospital of the Guangzhou Medical University concluded as following:

1. Agree to approve the scheme of the study, and the sponsor should carry out this study obeying the rules of the protocol strictly, and they should protect the rights of participants during the whole study.

2. If there are some updates of submitted materials, the sponsor should give them to the Ethics Committee in time. And when the study is carried out for a full year, the sponsor should present the research progress report to the Ethics committee. When the study is finished, please give the final report of the study to the Ethics committee.

Signature of chief: Jinping Zhen

Organization: the Ethics Committee in the First Affiliated Hospital of Guangzhou Medical University

Declaration: This Ethics Committee is an independent organization. The structure and the procedures of work are obeying the rules of GCP and the national laws. All the present members are in valid term.

Approval Form of Ethics Committee in the First Affiliated Hospital of Guangzhou Medical University (appendix)

No.201231

<table>
<tr><td>Project</td><td colspan="3">China National Epidemiological Study of Urolithiasis</td></tr>
<tr><td>Sponsor</td><td>Department of urology, The First Affiliated Hospital of Guangzhou Medical University</td><td>Head of this project</td><td>Guohua Zeng</td></tr>
<tr><td colspan="4">The lists of submitted materials and version number</td></tr>
<tr><td>No.</td><td>Name of document</td><td>Version</td><td>Date of version</td></tr>
<tr><td>1.</td><td>Ethics application form</td><td></td><td></td></tr>
<tr><td>2.</td><td>Ethics approval form</td><td></td><td></td></tr>
<tr><td>3.</td><td>The protocol of this study</td><td></td><td></td></tr>
<tr><td>4.</td><td>Informed consent (in Chinese)</td><td></td><td></td></tr>
<tr><td colspan="4"></td></tr>
<tr><td colspan="4"></td></tr>
<tr><td colspan="4"></td></tr>
<tr><td colspan="4">Consider the previous comments of the study, the Ethics Committee has made a copy of the submitted materials about this study, and agree to approve the study of "China National Epidemiological Study of Urolithiasis".
The sponsor should carry out this study obeying the protocol strictly, and they should protect the rights of participants during the study. If there are some updates of submitted materials, the sponsor should give them to the Ethics Committee in time.

Signature of chief
Date</td></tr>
</table>
